# Study on the correlation between the ultrasound phenotype and copy number variation of abnormal embryo in spontaneous abortion

**DOI:** 10.1111/jog.14987

**Published:** 2021-09-27

**Authors:** Shuyin Tan, Pingshan Pan, Zuojian Yang, Jiasun Su, Hongwei Wei

**Affiliations:** ^1^ Department of Clinical Genetics Maternal and Child Health Hospital of Guangxi Zhuang Autonomous Region Nanning China

**Keywords:** abnormal embryo, copy number variation, spontaneous abortion, ultrasound phenotype

## Abstract

**Objective:**

This study aimed to explore the correlation between the ultrasound phenotype and copy number variation (CNV) of abnormal embryos in spontaneous abortion by investigating the abnormal chromosome copy number of embryos at different developmental stages in early spontaneous abortion.

**Methods:**

A total of 539 patients who had early spontaneous abortion in our hospital between 2015 and 2019 were divided into seven groups according to the phenotype of abnormal embryonic development during pregnancy, and the embryonic tissues of the patients were subjected by single nucleotide polymorphism (SNP) microarray.

**Results:**

Among 377 cases with abnormal CNV, 295 (78.25%) cases had chromosome trisomy, and 28 (7.43%) cases had a combination of more than two chromosomes. Triploidy, tetraploidy, chromosome microdeletion/duplication, uniparental disomy, and monosomy 45,X were found in 32 (8.48%), five (1.32%), 31 (8.22%), four (1.02%), and eight (2.12%) cases, respectively. Two (0.53%) cases had autosomal chromosome 21 monosomy. Normal karyotype had the highest proportion in the empty sac group; trisomy 16 accounted for the bulk of chromosomes in the normal yolk sac group, complex triploidy occupied the most part in the abnormal yolk sac group, and no remarkable difference in chromosomal abnormality proportion was found between the normal and abnormal yolk sac groups; the most frequent chromosomal anomaly in a group of germ without cardiac activity and germ<5 mm was trisomy 16; triploidy was the most common in the group with 5 mm ≤ germ ≤ 15 mm, whereas the main distribution of chromosome karyotype was normal, followed by trisomy 13 in a group with germ>15 mm.

**Conclusion:**

Abnormal embryos with different ultrasound phenotypes in early spontaneous abortion have various CNV types and characteristic distribution. Thus, ultrasound combined with SNP array can provide a basis for the etiological analysis of embryos in spontaneous abortion.

## Introduction

Early spontaneous abortion is defined as miscarriage before 12 weeks of pregnancy, which can be a result of abnormal embryonic development. Spontaneous abortion occurs in approximately 15%–20% of pregnancies,^1^ and early spontaneous abortion accounts for 80% of spontaneous abortions.

The abnormal developmental morphologies of embryos in the early state can be described and revealed with ultrasound technology. The four ultrasonic characteristics during early pregnancy include gestational sac, yolk sac, germ, and cardiac activity.^2^, ^3^ These characteristics are the key indicators used to evaluate embryonic development. Abnormal early embryonic development, such as the absence of yolk sac and embryo (empty gestational sac), yolk sac without germ, yolk sac with germ but without cardiac activity, and yolk sac with germ and cardiac activity but with cardiac activity arrest, can occur at any stage and manifest in sonographic features, which are used to predict adverse outcome in the first trimester.^4^

The clinical manifestations of 50%–70% of chromosomal abnormalities in human embryos are anomalous or stagnant at the early phase of embryo formation.^5^ Fetal dysplasia has various ultrasound phenotypes. This study explored whether copy number variations (CNVs) and ultrasound abnormalities are correlated.

## Materials and Methods

Subjects in the Maternal and Child Health Hospital of Guangxi Zhuang Autonomous Region from January 2015 to December 2019 who underwent ultrasound examination and abortion villi detection by single nucleotide polymorphism (SNP) microarray for “abnormal or stagnant embryo” were retrospectively analyzed.

### Inclusion criteria

1. Termination of pregnancy was due to abnormal embryo development or embryo discontinuation at 6–12 weeks of gestation;
2. Gynecological ultrasound examinations during 6–8 weeks of pregnancy were ≥ 2 times;
3. Abortion tissues were tested by SNP microarray in the laboratory of our hospital.

### Exclusion criteria

1. Termination of pregnancy was not due to abnormal embryo development or embryo discontinuation;
2. Gynecological ultrasound examinations at 6–8 weeks of pregnancy were < 2 times;
3. Abortion tissues were not tested by SNP microarray.

### Diagnostic criteria for abnormal embryo development or embryo discontinuation

1. Yolk sac or germ was not found in the gestational sac at the sixth week of gestation;
2. The embryonic length only reached 4 mm, and no cardiac activity was detected by ultrasound after 1 week, or embryonic length was greater than 7 mm but without cardiac activity;
3. Cardiac arrest occurred after the occurrence of cardiac activity.

### Diagnostic criteria for abnormal yolk sac

1. Yolk sac diameter ≤ 3 mm;
2. Yolk sac diameter ≥ 8 mm;
3. Irregular yolk sac or yolk sac calcification.

### Classification criteria and definition of embryo ultrasound

Embryo abnormalities were categorized into four groups as follows:

1. Empty gestational sac group: No yolk sac and germ were found in the gestational sac at 6 weeks of pregnancy.
2. Yolk sac without germ group: Yolk sac was detected in the gestational sac at 5–6 weeks, but no germ was found after 2 weeks. This group was further divided into two subgroups (normal yolk sac group and abnormal yolk sac group) according to yolk sac shape.
3. Germ without cardiac activity group: Germ length was <4 mm or >7 mm, and no cardiac activity was detected by ultrasound after 1 weeks.
4. Cardiac arrest group: Cardiac activity was detected after 6 weeks of gestation, then ultrasound proved that the cardiac activity disappeared at least two times. This group was further divided into three subgroups on the basis of germ length (germ < 5 mm group, 5 mm ≤germ ≤ 15 mm group, germ > 15 mm group).

### SNP microarray

#### Technical principle

Gene probes with known sequences are integrated on the solid surface, and a large number of labeled nucleic acid sequences in the tested biological cells or tissues are hybridized with the probe array. Rapid detection of gene information is realized by detecting the corresponding hybridization probes. QIAamp DNA Blood Mini Kit was used to extract the genomic DNA of abortion tissues according to the operation manual of the kit (Qiagen, Germany), the DNA was eluted with Tris–EDTA solution. The DNA concentration was ≥50 ng/μL as determined by spectrophotometry, and the optical density at 260/280 nm was between 1.7 and 1.9. At least 200 ng DNA (4 μL) was taken for single‐base extension and chip staining according to the operating procedure. Human CytoSNP‐12 Beadchip Kit (Illumina, USA), which contains about 300 000 detection sites, was used.

ISCAN scanning system was used to collect data, and Karyostudio software (Illumina, USA) was used to analyze the results. The chip results were divided into three categories according to clinical significance: pathogenic, benign, and variant of uncertain significance. The databases used include the Database of Genomic Variant, Database of Chromosomal Imbalance and Phenotype in Humans using ensemble Resources, Online Mendelian Inheritance in Man, and International Standard for Cytogenomic Arrays Consortium database.

#### Interpretation criteria for positive copy number

The detected results of spontaneous abortion tissue specimens were interpreted as pathogenic CNVs, including chromosome trisomy and complex trisomy (more than two chromosome trisomy), triploidy, tetraploidy, chromosomal monosomy, microdeletion or microduplication, uniparental disomy (UPD), and others.

Chi‐square analyses were used to test the positive rate of each group by PASW statistics 18 software, *p* < 0.05 was regarded as significant.

### Ultrasound instrument and pregnancy marker measurement

The ultrasound monitoring instruments used in the study were TOSHIBA‐A500A (Japan), voluson E8 (United States), and ALOKA‐ARIETTA 70 (Japan), and the probe frequency was 5.5–7 MHz. Bladder position was identified, then vaginal probe was inserted into the posterior fornix of the vagina and scanned in longitudinal, transverse, and oblique directions. All ultrasound measurements were performed by the same sonographer with CDFI license (the sonographer is the third author).

The mean value of two meridians represents the size of the gestational sac (one meridian is the maximum inner diameter, and the other meridian is the maximum distance perpendicular to the maximum inner diameter). The maximum distance from the inner edge of the sac represents the yolk sac diameter. The length of an embryo is the maximum distance between the head and buttock diameter. Cardiac activity is a pulsation signal visible to the naked eye or a blood flow signal detected under B‐scan ultrasound.

### Curettage and retention of abortion tissue

Clinicians asked the subjects to sign the consent form for uterine curettage and detection of abortion tissue SNPs by microarray for the diagnosis of abnormal embryo development or embryo discontinuation. Bladder position was identified. Then, a suction tube with a negative pressure of 300–500 mmHg was used to suck the contents of the uterine cavity until rough. Villus tissue was selected, soaked in normal saline, and sent to the laboratory within 24 h. Decidual tissue was avoided in the whole process to prevent mixing.

## Results

A total of 539 cases met the diagnostic criteria of spontaneous abortion and successfully underwent chorionic chromosome examination in our hospital from January 2015 and December 2019. The mean maternal age was 32.8 ± 4.6 years (range 24–45 years), pregnancy times were 1–11, the number of births was 0–2, the average number of previous spontaneous abortion was 2.88 ± 1.76 (range 0–6), and the average gestational age at the moment of abortion was 7.75 weeks. The ultrasound phenotype, the number of cases (proportion), and the gestational age (M ± SD) of spontaneous abortion are shown in Table 1.

**TABLE 1:** Ultrasound phenotype and number of spontaneous abortion cases: Shows the number and proportion of spontaneous abortion patients with different ultrasound phenotypes

Ultrasound phenotype	Empty sac	Yolk sac without germ		Germ without cardiac activity	Cardiac arrest		
		Normal yolk sac	Abnormal yolk sac		Germ <5 mm	5 mm ≥ germ ≤ 15 mm	Germ > 15 mm
Cases (*n*)	75	56	45	169	40	102	52
Gestational age (*M* ± SD)	6.32 ± 0.71	6.80 ± 0.79	6.82 ± 0.65	7.17 ± 0.63	7.33 ± 0.75	7.79 ± 0.87	9.12 ± 0.56
Proportion (%)	13.91	10.38	8.34	31.35	7.42	18.92	9.64

*Note*: The horizontal is ultrasound phenotype, and the vertical is the number and proportion.

Among the 539 tissue samples collected, 162 (30.05%) showed no abnormality, and 377 (69.94%) cases were positive for abnormality.

Among the 377 cases with CNV abnormalities, 295 (78.25%) had trisomy, 32 (8.48%) had triploid, 31 (8.22%) had chromosome microdeletion/duplication, and 28 (7.43%) had a combination of more than two chromosomes. Additionally, sex chromosome monosomy, tetraploidy, and UPD (1–22, X) were found in eight (2.12%), five (1.32%), and four (1.06%) cases, respectively. Only two (0.53%) cases had autosomal chromosome 21 monosomy.

**FIGURE 1:**
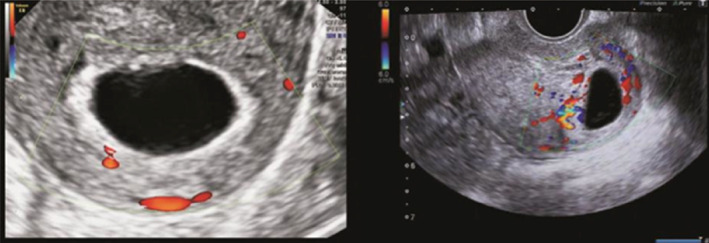
Empty sac group: Ultrasound pictures illustrating empty gestational sac on the 42nd day of pregnancy, and still empty gestational sac on the 53rd day of pregnancy. The copy number of abortion tissue detection was arr (1–22) x2, (X) x2

**FIGURE 2:**
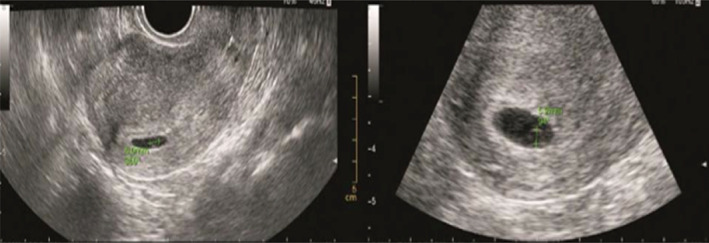
Yolk sac without germ group (normal yolk sac): Ultrasound pictures illustrating yolk sac with a diameter of 5 mm on the 39th day of pregnancy, and on the 49th day of pregnancy, ultrasound pictures illustrating yolk sac with a diameter of 5.2 mm but still without germ. The copy number of abortion tissue detection was arr (4) x3

**FIGURE 3:**
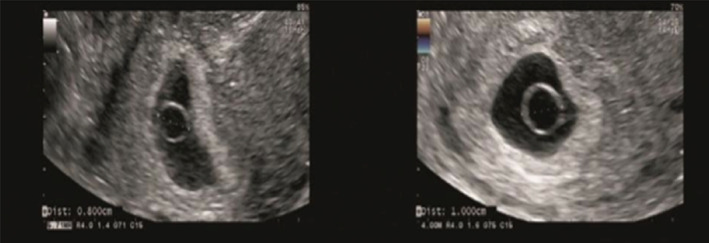
Yolk sac without germ group (abnormal yolk sac): Ultrasound pictures illustrating yolk sac with a diameter of 8 mm on the 49th day of pregnancy, and on the 55th day of pregnancy, ultrasound pictures illustrating yolk sac with a diameter of 10 mm but still without germ. The copy number of abortion tissue detection was arr (16) x3

**FIGURE 4:**
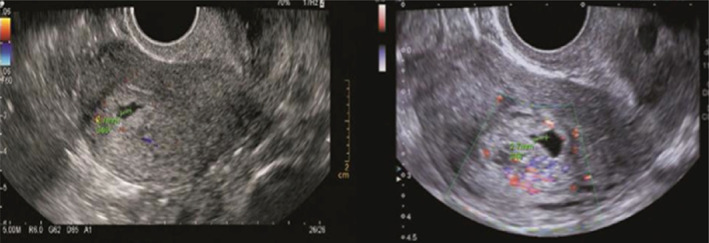
Germ without cardiac activity group: Ultrasound pictures illustrating the germ without cardiac activity was 2.7 mm at 6 weeks of gestation, and at 7 weeks of gestation, ultrasound pictures illustrating the germ without cardiac activity still was 2.7 mm. The copy number of abortion tissue detection was arr (20) x3

**FIGURE 5:**
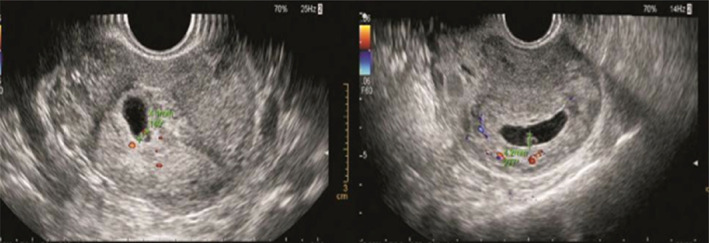
Cardiac arrest group (germ<5 mm): Ultrasound pictures illustrating the germ with cardiac activity was 4.1 mm on the 47th day of pregnancy, but on the 55th day of pregnancy, ultrasound pictures illustrating the cardiac activity disappeared and the germ was 4.2 mm. The copy number of abortion tissue detection was arr (22) x3 combined with arr (13) x3

**FIGURE 6:**
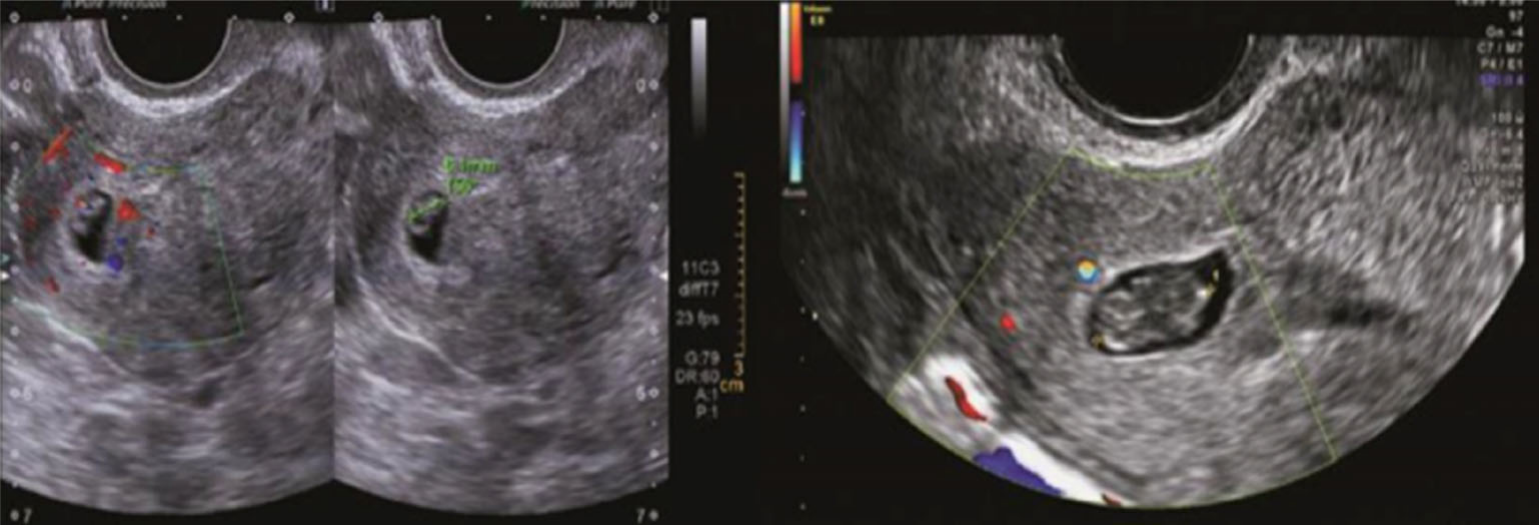
Cardiac arrest group (5 mm ≥ germ≤15 mm): Ultrasound pictures illustrating the germ was 6.1 mm and cardiac activity appeared on the 45th day of pregnancy, but on the 58th day of pregnancy, ultrasound pictures illustrating the cardiac activity disappeared and the germ was 14 mm. The copy number of abortion tissue detection was arr (1–22) x3, (X) x2, (Y) x1

**FIGURE 7:**
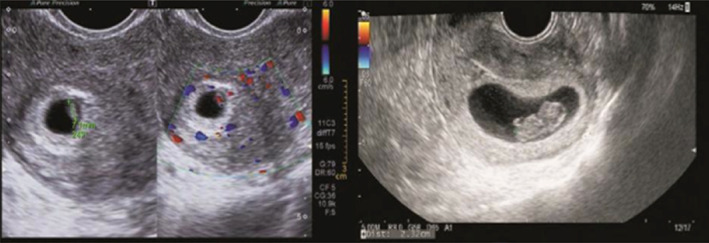
Cardiac arrest group (germ > 15 mm): Ultrasound pictures illustrating the germ was 7.1 mm and cardiac activity appeared on the 46th day of gestation, but on the 63th day of gestation, ultrasound pictures illustrating the cardiac activity disappeared and the germ was 23 mm. The copy number of abortion tissue detection was arr (13) x3

In this study, the ultrasound phenotypes of abnormal embryo development in spontaneous abortion were divided into seven groups. Each group had different CNVs. Their distribution is shown in Table 2. The ultrasound pictures and cases of the seven groups are shown in Figures 1, 2, 3, 4, 5, 6, 7.

**TABLE 2:** Ultrasound phenotype and abnormal copy number distribution of abnormal embryo in spontaneous abortion: Shows the characteristic distribution of different CNVs in various ultrasound phenotypes

Karyotype	Empty sac	Yolk sac without germ		Germ without cardiac activity	Cardiac arrest		
		Normal yolk sac	Abnormal yolk sac		Germ <5 mm	5 mm ≤ germ ≤ 15 mm	Germ > 15 mm
Normal copy number							
46,XY	22	3	4	17	4	18	12
46,XX	20	7	4	20	2	12	17
Autosomal copy number abnormality							
Autosomal trisomy	13	39	22	109	24	45	15
Autosomal monosomy	0	0	0	0	2	0	0
Triploidy	3	0	0	5	3	21	0
Tetraploidy	2	0	0	2		1	0
Compound trisomy	3	3	13	6	3	0	0
Microdeletion/duplication	10	4	1	9	2	3	2
UPD	2	0	1	1	0	0	0
Abnormal copy number of sex chromosome							
45, X	0	0	0	0	0	2	6
Total abnormal CNV	33	46	37	132	34	72	23
CNV‐positive rate	44.00%	82.14%	82.22%	78.11%	85.00%	70.59%	44.23%

*Note*: Chi‐square analyses of multiple samples were used to test the positive rate of the seven groups, *p* < 0.01 was regarded as significant. Data are *n* unless otherwise specified.

The distribution, number and proportion of each autosomal trisomy in seven groups of ultrasound phenotypes in this study are shown in Table 3. No cases of trisomy 1 and trisomy 19 were found in the study.

**TABLE 3:** Distribution, number, and proportion of each autosomal trisomy: Shows the characteristic distribution, number, and proportion of each autosomal trisomy in seven groups of ultrasound phenotypes in this study

Karyotype	Total (*n*)	Proportion (%)	Empty sac	Yolk sac without germ		Germ without cardiac activity	Cardiac arrest		
				Normal yolk sac	Abnormal yolk sac		Germ < 5 mm	5 mm ≥ germ ≤ 15 mm	Germ > 15 mm
+2	4	1.49	1	0	0	3	0	0	0
+3	10	3.75	0	3	0	6	1	0	0
+4	12	4.49	1	1	0	6	2	2	0
+5	3	1.12	0	0	0	0	0	3	0
+6	6	2.25	0	0	0	6	0	0	0
+7	11	4.12	1	2	2	3	1	2	0
+8	3	1.12	0	0	0	0	2	1	0
+9	7	2.62	3	0	2	2	0	0	0
+10	7	2.62	0	2	1	2	1	1	0
+11	2	0.75	0	0	1	1	0	0	0
+12	4	1.49	0	0	0	2	1	1	0
+13	10	3.75	0	1	0	0	0	5	4
+14	7	2.62	2	0	0	4	0	1	0
+15	22	8.24	1	2	2	3	1	9	4
+16	81	30.34	2	16	9	41	7	6	0
+17	5	1.87	0	0	2	3	0	0	0
+18	3	1.12	0	1	0	0	0	0	2
+20	8	2.99	0	0	3	3	2	0	0
+21	12	4.49	0	0	0	2	1	4	5
+22	50	18.73	2	11	0	22	5	10	0

*Note*: No cases of trisomy 1 and trisomy 19 were found in the study.

## Discussion

The development of early embryos can stop at different stages.^6^ Differences in the stage of embryonic apoptosis may be due to difference in chromosomal abnormalities,^7^ which carry different teratogenic and lethal genes. Therefore, an in‐depth study will help to further reveal the relationship between different chromosomal abnormalities and fetal development defects. This study is devoted to exploring the correlation between abnormal embryonic development in early pregnancy and chromosomal copy number abnormalities.

In this study, the chromosomal abnormalities of embryos in early spontaneous abortion manifested as abnormal chromosome number.^8^, ^9^ This finding is consistent with most research reports.^10^, ^11^ The ratio of chromosomal aneuploidy, including chromosome trisomy and compound trisomy, was the highest, followed by triploidy, CNV, tetraploidy, and few UPD and chromosomal monosomy. The most common type of ultrasound phenotypes of spontaneous abortion was found in the germ without cardiac activity group, followed by 5 mm ≤ germ ≤15 mm in the cardiac arrest group, and the empty sac group.

Empty sac accounted for 13.91% of the ultrasound phenotypes in this study. In the CNV examination of abortion tissues, the rate of normal chromosome was 56%. The empty sac group had the highest proportion of normal karyotypes among the different embryonic abnormalities. Consistent with the research reports of many scholars,^12^, ^13^ non‐genetic factors, such as immune abnormality, may play an essential role in the cause of empty gestational sac. The absence of embryonic tissue in an empty gestational sac was not ruled out. Relevant immune antibody test is recommended after embryo abortion, particularly those with empty yolk sac, even in first‐time spontaneous abortion.

No remarkable difference was found in the positive rate of chromosomal abnormalities between the abnormal and normal yolk sac groups. Our finding is inconsistent with a previous study,^14^ which reported the rate of chromosomal abnormality with abnormal yolk sac was higher than that with normal yolk sac. The present study suggested that the types of chromosomal abnormalities were different between the normal and abnormal yolk sac groups. The ratio of trisomy 16 was the highest in the normal yolk sac group, followed by trisomy 22 and normal chromosome karyotype, whereas triploidy accounted for the highest proportion in the abnormal yolk sac group, followed by trisomy 16. This difference may be related to the fact that complex trisomy contains more abnormal genes and thus results in abnormal yolk sac formation. Studies by other scholars suggested that trisomy 22^10^ or trisomy 16 accounts for the largest proportion when the yolk sac is larger than 5 mm.^15^ Our result is different from these studies.

The germ without cardiac activity group was the largest group in ultrasound phenotypes, and trisomy 16 had the highest percentage in this group, followed by trisomy 22 and normal karyotype. Such abnormal chromosome distribution was the same as that in the cardiac arrest group with germ <5 mm. This result is consistent with many previous literature reports.^16^ This result indicates that chromosomes 16 and 22 contain important regulatory genes for embryonic development after germ emergence. The cardiac tube system of the embryo cannot form normally or stops from developing soon after the appearance of cardiac activity when abnormalities occur.

In the cardiac arrest group with 5 mm ≤ germ ≤15 mm, triploidy had the largest ratio among abnormal karyotypes and accounted for 8.23% of all abortion tissues in this study. Compared with autosomal trisomy or monosomy, triploidy has greater developmental potential; embryos with longer germ length and even some trisomies could survive until the middle and late stages but with severe fetal growth restriction and other structural abnormalities as observed by ultrasound.^17^ This longer survival time may be because triploids do not cause the deletion of key genes related to early embryonic development.

In the cardiac arrest group with germ >15 mm, the distribution of chromosomal karyotypes according to proportion was as follows: normal karyotype, trisomy 13, trisomy 21, and trisomy 18, which was close to the characteristics of fetal chromosomal abnormalities in the second trimester.^18^ Our results showed that the trisomies of chromosomes 13, 18, and 21 have no substantial influence on early embryonic cell apoptosis.

Thirty‐one cases of chromosome microdeletion or microduplication were analyzed in this study. The proportion of these cases is 8.23%, which is slightly higher than those in other studies.^19^, ^20^ Among the 31 cases, 28 cases have more than 5 MB CNV, which exist in all chromosomes except for chromosome 21 and sex chromosome, for the above 28 cases, chromosome 9, chromosome 5 and chromosome 8 could be found in seven, five, and four cases. In addition, this study also found 10 cases have two chromosomes microdeletion or microduplication at the same time, in the subsequent karyotype verification of couples, it turned out that one side of the couple has chromosomal balanced translocation in six couples, which prompted that chromosomal balanced translocation was one of the main causes of recurrent abortion. Many studies have reported the relationship of pathogenic CNVs to early miscarriage and embryo termination,^21^ these studies indicated that CNVs are the causes of early miscarriage in patients. The findings greatly improve the diagnostic level and enrich the etiology database of spontaneous abortion.

Four cases of chromosome‐wide UPD were found in this study. Among them, two cases occurred in the empty pregnancy sac group. Chromosome‐wide UPD caused by parthenogenesis or parthenogenesis is one of the reasons for early spontaneous abortion.^22^, ^23^ Only two cases of autosomal chromosome 21 monosomy were found in the present study. Monosomy is lethal because of the large loss of genetic material; thus, spontaneous abortions caused by monosomy often occur very early in pregnancy.^24^

Monosomy 45,X, which accounted for 2.06% of the chromosomal abnormalities, was the only abnormality about sex chromosomes in this study, and its ultrasound phenotype was concentrated in the group with germ >15 mm. No additional CNV of sex chromosome was observed.^25^ Thus, chromosome gene loss caused by sex chromosome abnormality usually leads to miscarriage after 8 weeks of pregnancy and deformity, such as fetal edema.^26^

There were five cases of tetraploidy, the detection of tetraploidy by SNP‐array in this study was determined by calculating the frequency of B allele. So, we were unable to identify whether the formation mechanism of tetraploidy was the hidden twins or caused by nuclear mitosis, we also cannot rule out the existence of balanced(2:2)tetraploidy in normal group.

This study demonstrates that abnormal embryos at different development stages have specific ultrasound phenotypes,^27^ and different ultrasound phenotypes have different CNV features.^28^ The results can provide etiological basis for the diagnosis and treatments of spontaneous abortion, complement and perfect various embryonic development phenotype with abnormal CNV, improve the diagnosis and treatment of spontaneous abortion and clinical consultation level, and promote reproductive health. The weakness of our study is that the number of cases in some groups is insufficient; the limited number is not fully representative of the CNV characteristics of abnormal embryos.

## Author Contributions

Shuyin Tan: Conception and design, statistical analysis, collection and analysis of date, manuscript drafting/editing, writing—review and editing etc. Pingshan Pan, Jiasun Su, and Zuojian Yang: Material preparation, data collection and analysis, administrative technical support, etc. Hongwei Wei: Project development critically, funding acquisition, revised the work, etc. All authors commented on previous versions of the manuscript; all authors read and approved the final manuscript.

## Disclosure

No author has any potential conflict of interest.

## Data Availability

The data that support the findings of this study are available from the authors upon reasonable request. But we only have raw data, no data repository, no links.
